# Catalytic Degradation of Organic Dyes Induced by Tribo-Electrification Between Insulating Films

**DOI:** 10.3390/ma18102327

**Published:** 2025-05-16

**Authors:** Junhao Li, Xuefeng Xu

**Affiliations:** School of Technology, Beijing Forestry University, Beijing 100083, China; ljh229@bjfu.edu.cn

**Keywords:** methylene blue, contact–separation process, triboelectric effect, tribo-catalysis, radicals

## Abstract

In this study, a contact–separation triboelectric catalytic device was designed and constructed to systematically investigate the underlying degradation mechanism. The device enabled precise control of the contact–separation process between frictional surfaces. Polytetrafluoroethylene (PTFE) and polyethylene terephthalate (PET) films were selected as the triboelectric pair, and methylene blue (MB) was used as the model organic pollutant. Experimental results demonstrated that the contact–separation process in an aqueous environment effectively promotes the degradation of organic dyes. For an MB solution with an initial concentration of 5 mg/L, a degradation efficiency of 40.34% was achieved within 3 h. Moreover, the device exhibited excellent repeatability and stability, with no significant decline in performance after 15 h of continuous operation. Control experiments confirmed that the degradation originates specifically from the contact–separation interaction between the PTFE and PET surfaces. Free radical quenching experiments identified superoxide radicals (·O_2_^−^) and hydroxyl radicals (·OH) as the primary reactive species responsible for degradation. Based on these findings, a microscopic mechanism is proposed: during contact, triboelectric charging generates electrons (e^−^) and holes (h^+^) on the surfaces; upon separation, these charges interact with the solution—e^−^ reduce dissolved oxygen to form ·O_2_^−^, while h^+^ oxidize hydroxide ions (OH^−^) to produce ·OH. The combined action of ·O_2_^−^ and ·OH ultimately results in the efficient degradation of MB.

## 1. Introduction

China is the world’s largest exporter of dyes, accounting for more than 50% of the world’s total dye production. However, the dye production process comes with serious environmental pollution problems. Studies have shown that for every 1 ton of dye produced, about 744 cubic meters of wastewater will be discharged [1]. With the rapid increase in the discharge of dye wastewater, a large number of refractory organic pollutants are discharged into the water body, causing serious water pollution problems [2]. In order to solve this problem, commonly used treatment methods include oxidative decomposition [3,4], electrochemical decomposition [5,6], photocatalysis [7,8], and piezoelectric catalysis [9,10]. However, these methods have some limitations. For example, the strong oxidants used in the oxidative decomposition method can easily cause secondary pollution to water bodies [11]. The electrochemical decomposition method requires supporting power equipment, which has high energy consumption, and the electrode can get corroded easily [12]. The practical application of photocatalysis is limited by its low light utilization rate and inability to work in dark environments [13,14]. Piezoelectric catalysis requires ultrasonic-induced piezoelectric materials to produce piezoelectric effects, resulting in poor environmental adaptability [14]. Therefore, it is of great significance to find a method with high efficiency, low energy consumption, environmental friendliness, and strong applicability for organic water pollution.

Tribo-electrification (also known as contact electrification) refers to the phenomenon of electrification on the surface of two materials after contact, and it is widely present in nature and production and life [15,16]. Tribo-electrification has been widely used in triboelectric nanogenerators [17,18], electrostatic precipitators [19,20], and sensors [21,22]. Since Kajdas et al. [23] first proposed the concept of tribo-catalysis, the degradation of organic dyes by tribo-catalysis has become an emerging research direction. In early studies, tribo-catalytic degradation of dyes was usually done by magnetic stirring. For example, Li et al. [24] successfully achieved efficient degradation of rhodamine B dye by adding Ba0.75Sr0.2TiO3 (BST) nanoparticles to rhodamine B (RhB) dye solution and using a polytetrafluoroethylene (PTFE) stir bar for magnetic stirring. After 12 h of magnetic stirring, the degradation rate of the dye is as high as 99%. They believe that this degradation is due to the collision between the stirrer and the nanoparticles during the stirring process, resulting in the transfer of charge on the surface of the two sides, resulting in the formation of electron-hole pairs, inducing a series of redox reactions, and finally achieving the degradation of organic dyes. Follow-up studies have shown that BiOIO3 nanoparticles and PTFE stir bar [25,26], Ba4Nd2Fe2Nb8O30 (BNFN) submicron powders and PTFE stir bars [26], and TiO2 nanoparticles and PTFE stir bars [27] can significantly degrade RhB dyes. Wu et al. [28] further demonstrated that even friction between single materials can effectively degrade organic dyes. They found that after 12 h of PTFE magnetic bar agitation, PTFE particles degraded RhB by more than 90%. Recently, Wang et al. [29] placed a methyl orange (MO) solution mixed with fluorinated ethylene propylene copolymer (FEP) powder in an ultrasonic device and completely degraded the MO dye at an initial concentration of 5 mg/L after 180 min of sonication. They believe that the electrons transferred between the dielectric powder and water can directly catalyze the reaction without the use of conventional catalysts, a process called contact-electro-catalysis.

Despite the advantages of tribo-catalysis, such as a wide range of mechanical energy sources and a wide selection of friction materials, there are some key challenges to existing technologies. First, most commonly used tribo-electric materials are in the form of nanoparticles or powders, which are difficult to recover and may cause secondary pollution to water bodies, thereby limiting their application in open aquatic environments. Second, existing tribo-catalytic excitation methods, such as magnetic stirring and ultrasonic treatment, are challenging to implement under natural environmental conditions, which significantly restricts the practical deployment of this technology. Furthermore, the amount of charge generated by the tribo-electric effect is relatively low and can be influenced by environmental factors such as humidity and surface condition of materials, leading to catalytic efficiency that is often less stable compared to conventional techniques. Therefore, it is important to explore new excitation methods and friction material forms to expand the application range of tribocatalysis. In this paper, a contact–separation tribo-electric catalytic device was developed, and PTFE and PET films were used as friction pair materials, and the degradation of methylene blue (MB) dyes was experimentally studied during the contact–separation process between the films. The catalytic mechanism of contact–separation tribo-catalysis was further analyzed by comparative experiments and free radical quenching experiments. This study not only verifies the feasibility of tribo-catalysis in the degradation of organic pollutants, but also provides a new idea for the development of efficient and environmentally friendly water pollution control technologies.

## 2. Materials and Methods

### 2.1. Reagents and Materials

In this study, methylene blue (MB) was selected as the target dye to be degraded. MB is a commonly used dye and indicator in a wide range of applications for biological staining, chemical analysis, and water contamination treatment studies. The MB used in the experiment was analytically pure and was purchased from Shanghai Aladdin Reagent Company. Methyl orange (MO) is another dye to be degraded, the purity is analytical pure, purchased from Shanghai Aladdin Company. In addition, disodium ethylenediaminetetraacetic acid (EDTA-2Na), isopropanol (IPA), potassium bromate (KBrO_3_), and p-benzoquinone (BQ) are used as free radical quenchers, all of which are analytically pure, and are also purchased from Shanghai Aladdin Reagent Company. Anhydrous ethanol (analytically pure) is used to clean the surface of friction materials and was purchased from Shanghai Jizhi Biochemical Technology Co., Ltd. The friction materials are 0.05 mm thick polyethylene terephthalate (PET) film and polytetrafluoroethylene (PTFE) film, which are purchased from Taizhou Chenguang Plastics.

### 2.2. Experimental Setup

**Figure 1:**
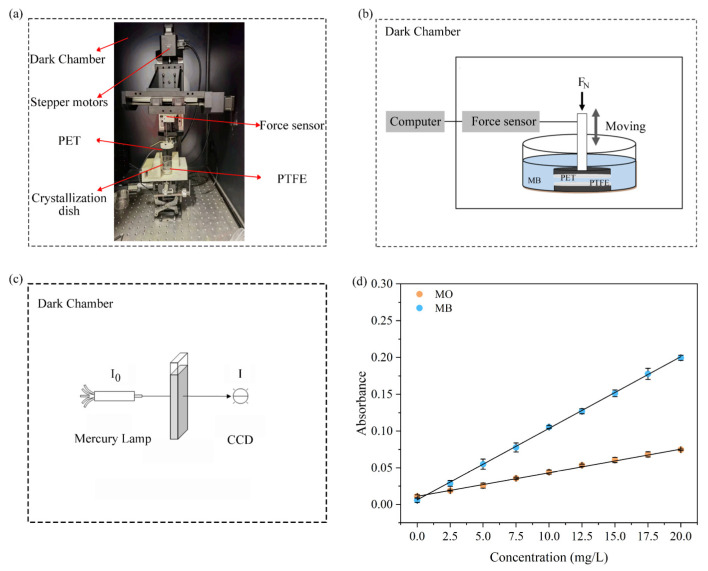
(**a**) Photograph of the contact–separation tribo-catalytic device. (**b**) Schematic diagram of the contact–separation tribo-catalytic process. (**c**) Schematic diagram of the absorbance measurement device. (**d**) Relationship between the absorbance and concentration of MB and MO solutions.

The contact–separation tribo-catalytic device is mainly composed of a mechanical system and a control system (see Figure 1a). The mechanical system uses a stepper motor and a lead screw to achieve contact and separation of the upper and lower friction materials. The control system is equipped with a K3D40 3D force sensor (0–2 N with 0.5% accuracy, purchased from ME-Messsyteme, Brandenburg, Germany) for real-time monitoring and precise control of the load during the experiment. In order to avoid the influence of ambient light on the experiment, the entire device was placed inside a blackout box.

### 2.3. Experimental Methods

During the experiment, the PTFE film was affixed to the bottom of a crystallizing dish to act as the lower tribo-electric layer. Subsequently, 30 mL of a 5 mg/L methylene blue (MB) solution was introduced into the dish. The PET film, serving as the upper tribo-electric material, was driven by a stepper motor to perform vertical contact–separation motion with the PTFE film in the solution (see Figure 1b). In the experiment, the contact pressure was set to 0.4 N, the contact time was 3 s, the separation time was 5 s, and the separation distance was 1.25 mm. The total duration of a single experiment was 3 h, and 0.35 mL of MB solution was sampled at 1 h intervals, and the degradation rate of the solution at different times was measured to evaluate the catalytic effect of contact–separation tribo-electric activation.

The degradation rate of the solution was determined by measuring its absorbance. According to the Lambert–Beer Law, when a beam of monochromatic parallel light passes perpendicularly through a homogeneous, non-scattering absorbing medium of a certain thickness, the absorbance is directly proportional to the concentration of the absorbing species [30]. The relationship can be expressed as follows:

(1) A=lg(1/T)=kbc

where *A* is the absorbance, *T* is the transmittance (i.e., the ratio of the transmitted light intensity *I*_1_ to the incident light intensity *I*_0_), *k* is the molar absorptivity (absorption coefficient), *b* is the path length of the absorbing medium, and *c* is the concentration of the absorbing species. Based on the measured absorbance values, the degradation efficiency of the solution at various time points can be calculated.

In this study, the absorbance measurement system consisted of a mercury lamp as the light source and a CCD spectrometer for spectral detection (see Figure 1c). According to the changes in maximum absorbance (at 663 nm for MB and 464 nm for MO [31,32]), the experimental results demonstrated a strong linear correlation between absorbance and the concentrations of MB and MO solutions, as confirmed by linear fitting (see Figure 1d). The fitted relationships are as follows:

MB:

(2) A=0.0097c+0.0057

MO:

(3) A=0.0033c+0.0111

where *A* is the absorbance of the solution of the dye to be degraded, and *c* is the corresponding solution concentration (unit: mg/L). Based on these fitting formulas, the concentration of the dye solution to be degraded can be calculated by measuring the absorbance. Then, the degradation rate *D* of the solution is calculated, and its expression is as follows:

(4) $$D=\frac{c_{1}-c_{2}}{c_{1}}\times 100\%=\frac{A_{1}-A_{2}}{A_{1}-A_{0}}\times 100\%$$

where $c_{1}$ is the initial concentration of the solution, and $c_{2}$ is the concentration after the degradation of the solution. *A*_0_ is the absorbance corresponding to the initial concentration of zero, *A*_1_ is the absorbance corresponding to the initial concentration of the solution, and *A*_2_ is the absorbance after experimental degradation.

### 2.4. Quenching Experiment of Active Substances

In this study, p-benzoquinone (BQ), disodium ethylenediaminetetraacetic acid (EDTA-2Na), isopropanol (IPA), and potassium bromate (KBrO_3_) were selected as superoxide radicals (·O_2_^−^), hydroxyl radical (·OH), hole (h^+^), and electron (e^−^) quenchers [28,33,34]. In the experiment, 10 mL of IPA (99.5% purity) or 10 mL of BQ, EDTA-2Na, and KBrO_3_ at a concentration of 10 mg/L were added to the MB solution. By comparing the degradation rate of dye with or without quencher, the mechanism of different free radicals on dye degradation was analyzed.

## 3. Results and Discussion

### 3.1. Degradation Effect of PET and PTFE for Organic Dyes

**Figure 2:**
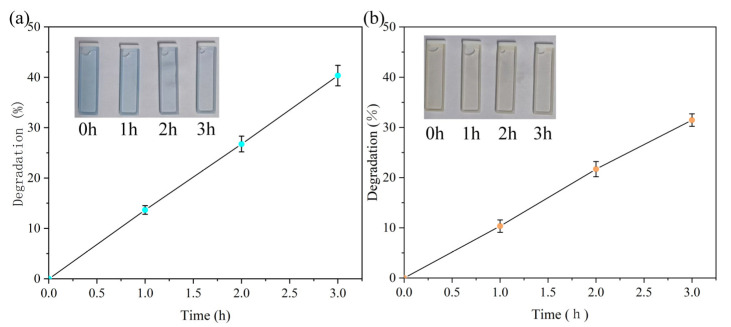
(**a**) Catalytic degradation of MB by PTFE and PET. (**b**) Catalytic degradation of MO by PTFE and PET.

The MB solution was initially concentrated at 5 mg/L and had a dark blue color (Figure 2a). As the contact–separation experiment progressed, the color of the MB solution gradually lightened, and the color of the solution became significantly lighter after 3 h (Figure 2a). This phenomenon shows that the concentration of the solution gradually decreases and the degradation rate gradually increases as the experiment progresses. By measuring the concentration of the solution at different moments and calculating the degradation rate, we found that the degradation rate of the solution increased linearly with time. After a 3 h contact–separation process, the degradation rate of the MB solution reached 40.34%. Experiments were carried out on the 5 mg/L MO solution, and it was found that the MO solution gradually faded as the contact separation progressed (Figure 2b). It was calculated that after 3 h of contact separation, the degradation rate of MO solution reached 31.46% (Figure 2b). This shows that the developed contact separation tribo-catalytic device has a degrading effect on a variety of dyes.

As shown in Table 1, the contact-separation-based tribo-catalysis developed in this study demonstrates a comparable degradation performance for methylene blue (MB) to other conventional degradation techniques. Although the degradation efficiency of tribocatalysis (40.34% within 180 min) is relatively lower than that of photodegradation, Fenton and photo-Fenton processes, catalytic ozonation, heterogeneous catalytic degradation, and electrocatalytic degradation, its advantages in environmental friendliness, low cost, and operational simplicity make it a promising candidate for specific application scenarios. Future research efforts could focus on optimizing friction materials, improving the contact–separation mechanism, and exploring synergistic combinations with other degradation technologies to enhance both degradation efficiency and practical applicability.

**Figure 3:**
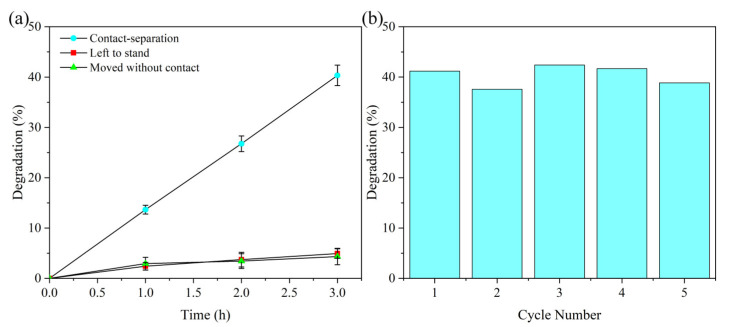
(**a**) Catalytic degradation of MB by PTFE and PET: experimental and control studies. (**b**) Time-dependent stability of catalytic degradation performance.

In order to explore the real cause of MB dye degradation, we designed and implemented a series of comparative experiments. First, the PET and PTFE films were placed in the MB solution, and after 3 h of standing, the degradation efficiency of the MB solution was only 4.93% (Figure 3a). Subsequently, the PET film was moved up and down in solution without coming into contact with PTFE, and the results showed that after a 3 h experiment, the degradation efficiency of MB was only 4.34% (Figure 3a). These experimental results suggest that the motion of the film material itself, as well as the film material and solution, is not the main cause of the efficient degradation observed earlier. In contrast, the efficient degradation of MB dyes should be mainly attributed to the contact–separation process between PET and PTFE films.

In the literature, the reaction rate constant (k) is often used to describe the degradation efficiency of a solution, which is defined as follows:

(5) $$k=-\ln(c/c_{0})/t$$

where $c_{0}$ is the initial solution concentration, c is the current solution concentration, and t is the reaction time [40]. The results show that the reaction rate constant k of the contact–separation process between PET and PTFE film is 0.1714 h^−^¹, while the k value of PET and PTFE is only 0.0165 h^−^¹ when standing, and 0.0166 h^−^¹ when PET is only moving up and down. This result further confirms that the efficient degradation of MB dyes is mainly due to the contact–separation process between PET and PTFE films.

The contact–separation process between thin film materials can effectively degrade MB dyes, which shows great application potential in solving the problem of dye contamination. Therefore, it is necessary to further evaluate the temporal stability of the tribocatalysis of PET and PTFE film materials. First, a catalytic degradation experiment was performed in 30 mL of MB solution at a concentration of 5 mg/L, and after 3 h, the absorbance of MB solution was measured, and its degradation efficiency was calculated. Subsequently, the MB solution on the surface of the film material was washed with absolute ethanol, and after the cleaning was complete, the film material was placed in a new MB solution for the next round of experiments. The results showed that the catalytic degradation rate of MB remained stable at around 40% after five cycles of experiments (Figure 3b). This result confirms that the tribo-catalytic effect of PTFE and PET film materials has high temporal stability.

### 3.2. Degradation of Free Radicals

Electrons (e^−^) react with oxygen in water to form superoxide radicals (·O_2_^−^), while holes (h^+^) react with water to form hydroxyl radicals (·OH). ·O_2_^−^ and ·OH exhibits strong oxidizing capacity in solution and are thought to be the main active substance responsible for dye decomposition [41,42,43]. In order to deeply analyze the mechanism of the contact–separation process between friction pairs on the degradation of MB in this paper, we designed a comparative experiment of free radical quenching.

**Figure 4:**
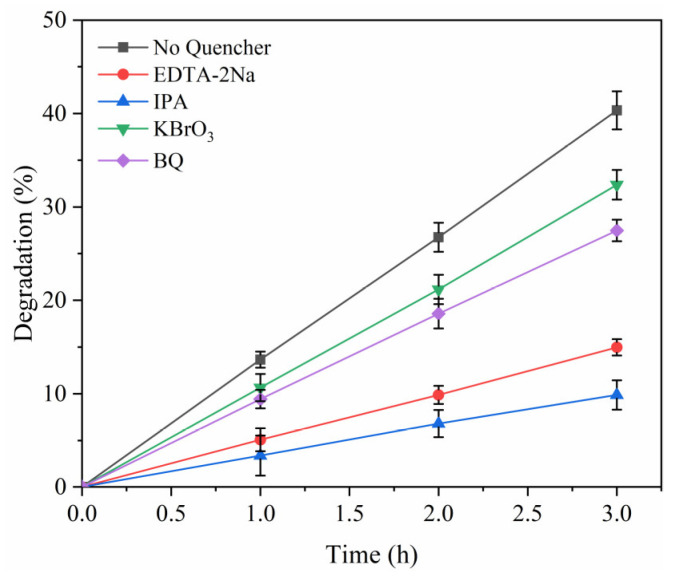
Results of free radical quenching comparative experiments.

In this study, we selected p-benzoquinone (BQ), ethylenediaminetetraacetic acid disodium (EDTA-2Na), isopropanol (IPA), and potassium bromate (KBrO_3_), respectively, as quenchers for ·O_2_^−^, ·OH, h^+^, and e^−^. By comparing the degradation rate of quencher with that without quencher, the mechanism of different free radicals on dye degradation was analyzed. The experimental results showed that the degradation rates of MB were 27.48% (BQ), 14.96% (EDTA-2Na), 9.88% (IPA), and 32.38% (KBrO_3_) after the addition of the above quencher (see Figure 4). Among them, IPA had the most significant inhibitory effect on MB degradation, indicating that ·OH plays a leading role in the degradation of MB. In addition, the introduction of BQ also inhibited the degradation of MB to a certain extent, indicating that ·O_2_^−^ radicals also contribute to the degradation process.

### 3.3. Contact Separation Tribocatalytic Mechanism

**Figure 5:**
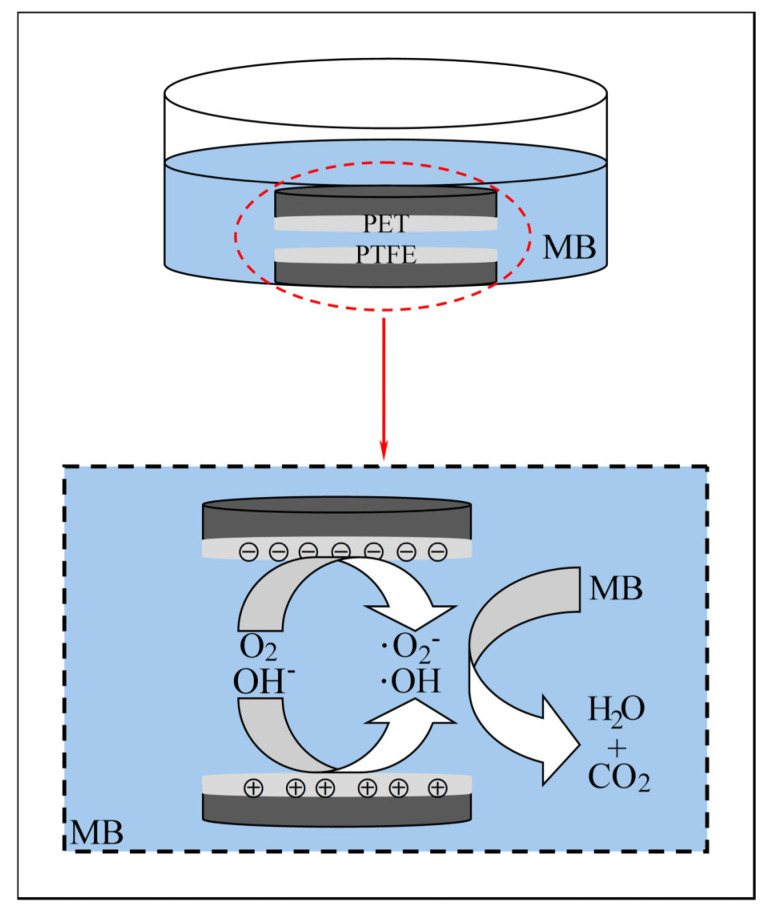
Contact–separation tribo-catalytic mechanism.

Based on the above experimental results, we propose a mechanism for the catalytic degradation of organic dyes during the contact–separation process of the friction pair. Experiments show that during the contact phase, electrons are transferred from the PET surface to the PTFE surface due to the difference in the work functions of the PET and PTFE surfaces, resulting in additional electrons (e^−^) and holes (h^+^) on the two surfaces, respectively. In the separation stage, the surface of the friction pair is in contact with the solution, and the electrons (e^−^) on the surface react with the oxygen in the water to form superoxide radicals (·O_2_^−^), while holes (h^+^) react with hydroxide ions (OH^−^) to form hydroxyl radicals (·OH). These free radicals (·O_2_^−^ and ·OH) reacts with methylene blue (MB), resulting in the degradation of MB (see Figure 5). The catalytic degradation reaction during the contact–separation process can be described by the following equation:

(1) Tribo-electric energizing:

(6) $$PET+PTFE\overset{\mathrm{Contact-separation}}{\to }PET+h^{+}+PTFE+e^{-}$$

(2) Free radical generation:

(7) $$e^{-}+O_{2}\to \cdot O_{2}^{-}$$

(8) $$h^{+}+OH^{-}\to \cdot OH$$

(3) Dye degradation:

(9) $$MB+\cdot O_{2}^{-}+\cdot OH\to Degradation$$

In summary, the contact–separation process induces charge transfer through tribo-electric induction, which in turn promotes the generation of reactive free radicals, thereby achieving effective catalytic degradation of organic dyes. This mechanism not only reveals the key role of triboelectrification in the degradation of organic pollutants but also provides an important theoretical basis for the subsequent optimization of the design of tribo-catalytic materials.

## 4. Conclusions

In this study, we designed and built a contact-separated tribo-catalytic device with PET and PTFE films as friction pairs, and successfully achieved efficient catalytic degradation of methylene blue (MB) dyes. The experimental results showed that the degradation rate of MB reached 40.34% in the reaction time of 3 h, and the catalytic degradation process showed good reproducibility and stability.

Through comparative experiments, we verified that the root cause of the catalytic action lies in the contact–separation behavior between PET and PTFE film materials. Further studies found that during the contact–separation process, the surface was charged to promote the formation of superoxide radicals in the solution (O_2_^−^) and hydroxyl radicals (·OH), these free radicals play a key role in the MB degradation process. Among them, hydroxyl radicals (OH) are the main active substance and contribute significantly to degradation.

This study not only reveals the important role of tribo-electrification in the catalytic degradation of organic dyes but also provides theoretical support and experimental basis for the development of new environmentally friendly catalytic technologies. This discovery provides an efficient, low-cost, and sustainable solution for dye wastewater treatment, and has broad application prospects. Future research can further optimize material selection, surface structure design, and reaction conditions to improve catalytic efficiency and expand its application in practical environmental management.

## Figures and Tables

**Table 1:** Degradation performance of methylene blue using different methods.

Degradation Method	Degradation (%)	Experimental Time (min)	Reference
photodegradation	94.4	120	[35]
Fenton and photo-Fenton	99.7	60	[36]
catalytic ozonation	84	20	[37]
heterogeneous catalytic degradation	nearly 100	120	[38]
electrocatalytic degradation	95.56	90	[39]
tribo-catalysis	40.34	180	this study

## Data Availability

The original contributions presented in the study are included in the article. Further inquiries may be directed to the corresponding author.
